# Prediction of pharmacokinetic/pharmacodynamic properties of aldosterone synthase inhibitors at drug discovery stage using an artificial intelligence-physiologically based pharmacokinetic model

**DOI:** 10.3389/fphar.2025.1578117

**Published:** 2025-04-28

**Authors:** Mengjun Zhang, Keheng Wu, Sihui Long, Xiong Jin, Bo Liu

**Affiliations:** ^1^ School of Chemical Engineering and Pharmacy, Wuhan Institute of Technology, Wuhan, China; ^2^ Yinghan Pharmaceutical Technology (Shanghai) Co., Ltd., Shanghai, China; ^3^ School of Chemistry and Chemical Engineering, Shanghai University of Engineering Science, Shanghai, China

**Keywords:** AI-PBPK, PK/PD simulation, CYP11B2, aldosterone synthase inhibitors, machine learning

## Abstract

The objective of this study is to develop an artificial intelligence-physiologically based pharmacokinetic (AI-PBPK) model to predict the pharmacokinetic (PK) and pharmacodynamic (PD) properties of aldosterone synthase inhibitors (ASIs), enabling selection of the right candidate with high potency and good selectivity at the drug discovery stage. On a web-based platform, an AI-PBPK model, integrating machine learning and a classical PBPK model for the PK simulation of ASIs, was developed. Baxdrostat, with the most clinical data available, was selected as the model compound. Following calibration and validation using published data, the model was applied to estimate the PK parameters of Baxdrostat, Dexfadrostat, Lorundrostat, BI689648, and the 11β-hydroxylase inhibitor LCI699. The PD of all five compounds was predicted based on plasma free drug concentrations. The results demonstrated that the PK/PD properties of an ASI could be inferred from its structural formula within a certain error range, providing a reference for early ASI lead compounds screening and optimization. Further validation and refinement of this model will enhance its predictive accuracy and expand its application in drug discovery.

## Graphical Abstract

**Figure:**
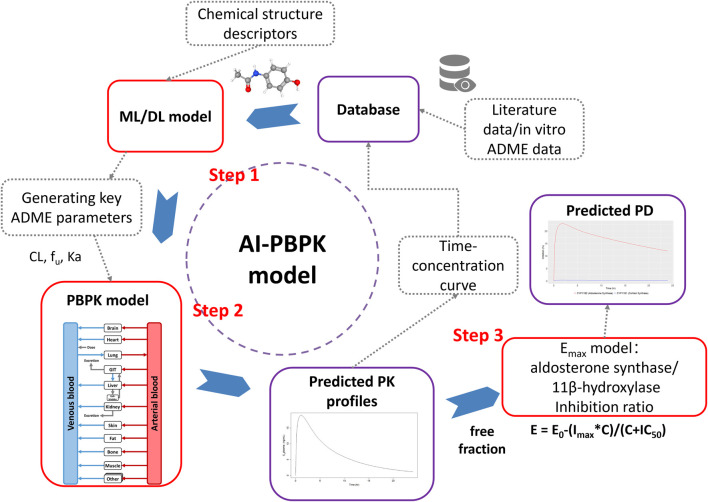
Steps for predicting a compound’s PK using the AI-PBPK model and predicting enzyme inhibition by combining it with the E_max_ model are shown. In Step 1, the compound’s structural formula is input into the AI model to generate key ADME parameters and physicochemical properties of the compound. In Step 2, these parameters are used in the PBPK model to predict PK profiles of the compound. In Step 3, a PD model is developed to predict the inhibition rate of aldosterone synthase and 11β-hydroxylase based on the plasma free concentration of the drug.

## 1 Introduction

**FIGURE 1:**
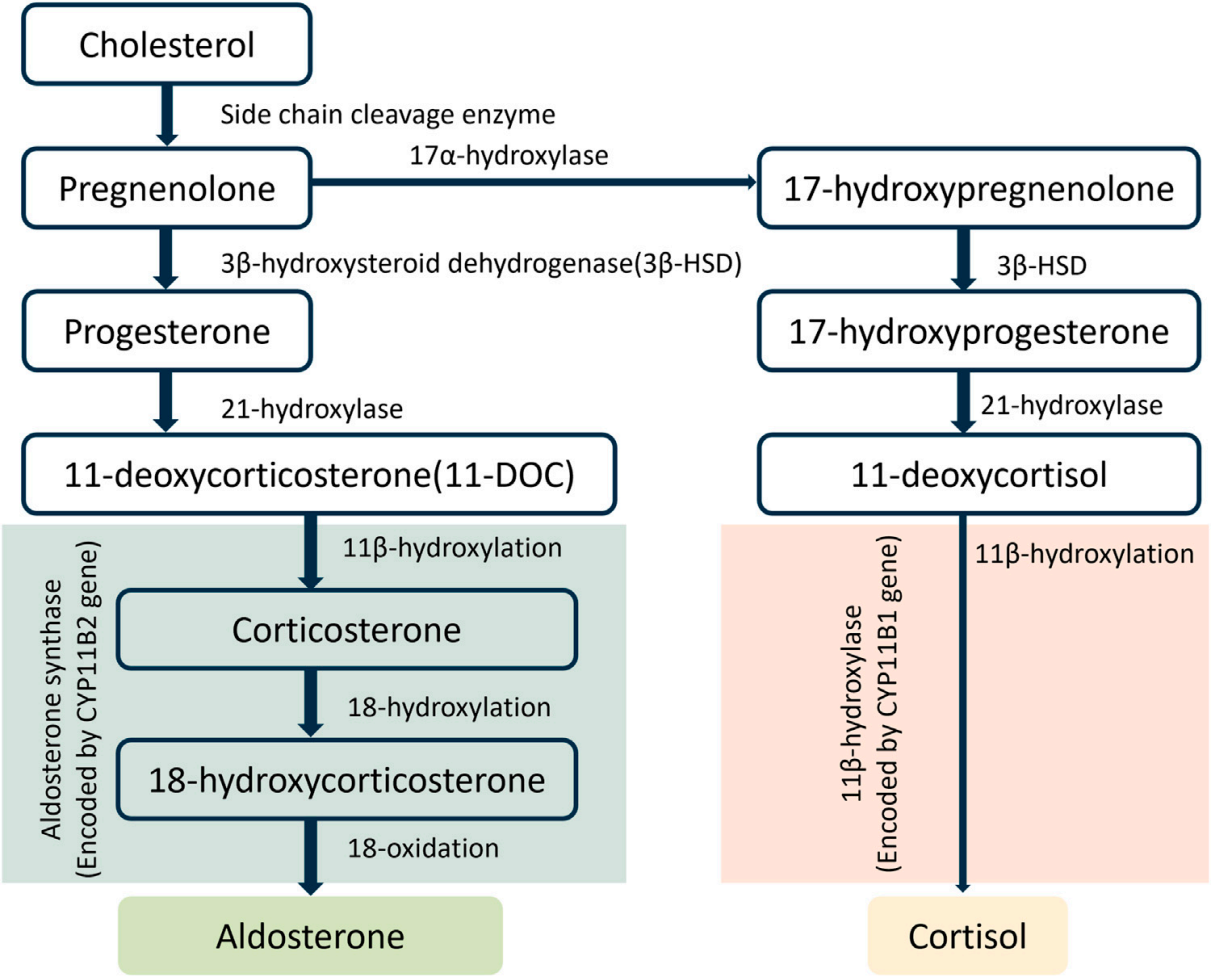
Biosynthetic pathways of aldosterone and cortisol.

Primary hyperaldosteronism, a common cause of resistant hypertension and an underlying cause of cardiac and renal diseases, is usually treated with mineralocorticoid receptor (MR) antagonists (Awosika et al., 2023; Miguel et al., 2024; Verma et al., 2024). However, these drugs are not always well tolerated and can cause a counterregulatory increase in aldosterone secretion, limiting their efficacy (Bogman et al., 2017). Aldosterone is synthesized from cholesterol through a series of enzymatic steps, with the last step catalyzed by aldosterone synthase (AS) which is encoded by the CYP11B2 gene (Figure 1). Researchers have targeted AS inhibition to curb the production of aldosterone (Andersen et al., 2012; Hargovan and Ferro, 2014). Yet, most aldosterone synthase inhibitors (ASIs) also exert an effect on 11β-hydroxylase. This enzyme, encoded by the CYP11B1 gene, facilitates the synthesis of cortisol, a steroid hormone structurally related to aldosterone (Nishimoto et al., 2023). This lack of selectivity of ASIs leads to side effects. Thus, compounds which selectively inhibit AS without affecting 11β-hydroxylase are needed. To identify an ideal ASI, a *de novo* design approach could be applied. But it can be extremely time-consuming and costly. A more feasible approach, i.e., comparison and optimization of the pharmacokinetic (PK) and pharmacodynamic (PD) properties and selectivity of existing ASIs (Mazzieri et al., 2024) might provide a solution. A drug’s PK/PD properties can be experimentally determined, as done conventionally. But again, this process usually is long and resource-intensive (Haid and Reichel, 2024). To shorten the drug discovery process, we developed an artificial intelligence (AI)-augmented physiologically based pharmacokinetic (PBPK) model to predict the PK and PD of ASIs.

A classical physiologically based pharmacokinetic model is well established, and it integrates physiological characteristics with a drug’s physicochemical properties to predict the drug’s behavior across different populations (Peters, 2021; Balhara et al., 2022). It helps optimize selection of the drug’s dosage and dosage form, as well as assessment of efficacy of the drug, by simulating its absorption, distribution, metabolism, and excretion (ADME) (Zou et al., 2020). Key input parameters for the classical PBPK model include human physiological parameters and molecule-specific parameters. To ensure accuracy of the classical PBPK model, comprehensive collection of the above parameters on software platforms such as GastroPlus and Simcyp are required (Arafat et al., 2021; Ezuruike et al., 2022). But at the drug discovery stage, molecule-specific parameters are often constrained by extensive *in vitro* experiments (Li et al., 2024). In recent years, the use of AI-based approaches to predict physiological parameters has emerged as a promising alternative. A number of web-based ADMET prediction tools have been developed, including ADMET-AI (Swanson et al., 2023), SwissADME (Daina et al., 2017), pkCSM (Azzam, 2023), XenoSite Web (Dang et al., 2020), and ADMETlab 3.0 (Fu et al., 2024). These tools offer advantages such as high efficiency, simplicity, and easy data interpretation by chemists. However, they have limitations such as single predictive function, incapability to predict PK and PD of compounds, and failure to derive physiologically relevant information from the structural formula of compounds.

To address these limitations, on our web-based B^2^O Simulator^®^ (B^2^O stands for Bioavailability, Bioequivalence, Optimization) platform, we developed a general AI-PBPK model, which integrates the PBPK model with machine learning (ML) and deep learning (DL), enabling a comprehensive prediction of a drug’s PK/PD profile from its molecular structure. At the drug discovery phase, when there are not enough drug-specific parameters to support the classical PBPK model, machine learning can be used to predict them based on the drug’s structural formula. With the aid of ML, the classical PBPK model can screen more potential candidate compounds at the preclinical drug discovery phase, avoiding unnecessary testing of a large number of candidates and shortening the process of advancing the candidates to preclinical studies. The AI-PBPK model facilitates the simulation and prediction of the behavior of diverse drug candidates *in vivo*. With it, we can better understand how drugs interact with the physiological systems, and predict their distribution, metabolism, and elimination in the body more efficiently (Chen et al., 2021; Chou and Lin, 2023), and reduce the dependence on experimental data at the screening phase. Therefore, the AI-PBPK model can have a broad perspective in facilitating drug safety assessment, efficacy prediction, formulation optimization, and therapy personalization (Marques et al., 2024; Visan and Negut, 2024).

In this study, an AI-PBPK model was built and used to predict the PK/PD properties of five ASIs, assisting the identification of the optimal candidate. In general, the predicted results are in good agreement with experimental observations, with occasional discordance, indicating the applicability of our model. With further improvement of the robustness of our AI-PBPK model, it is expected to be widely used in drug discovery.

## 2 Methods

### 2.1 Overall workflow of model building

**FIGURE 2:**
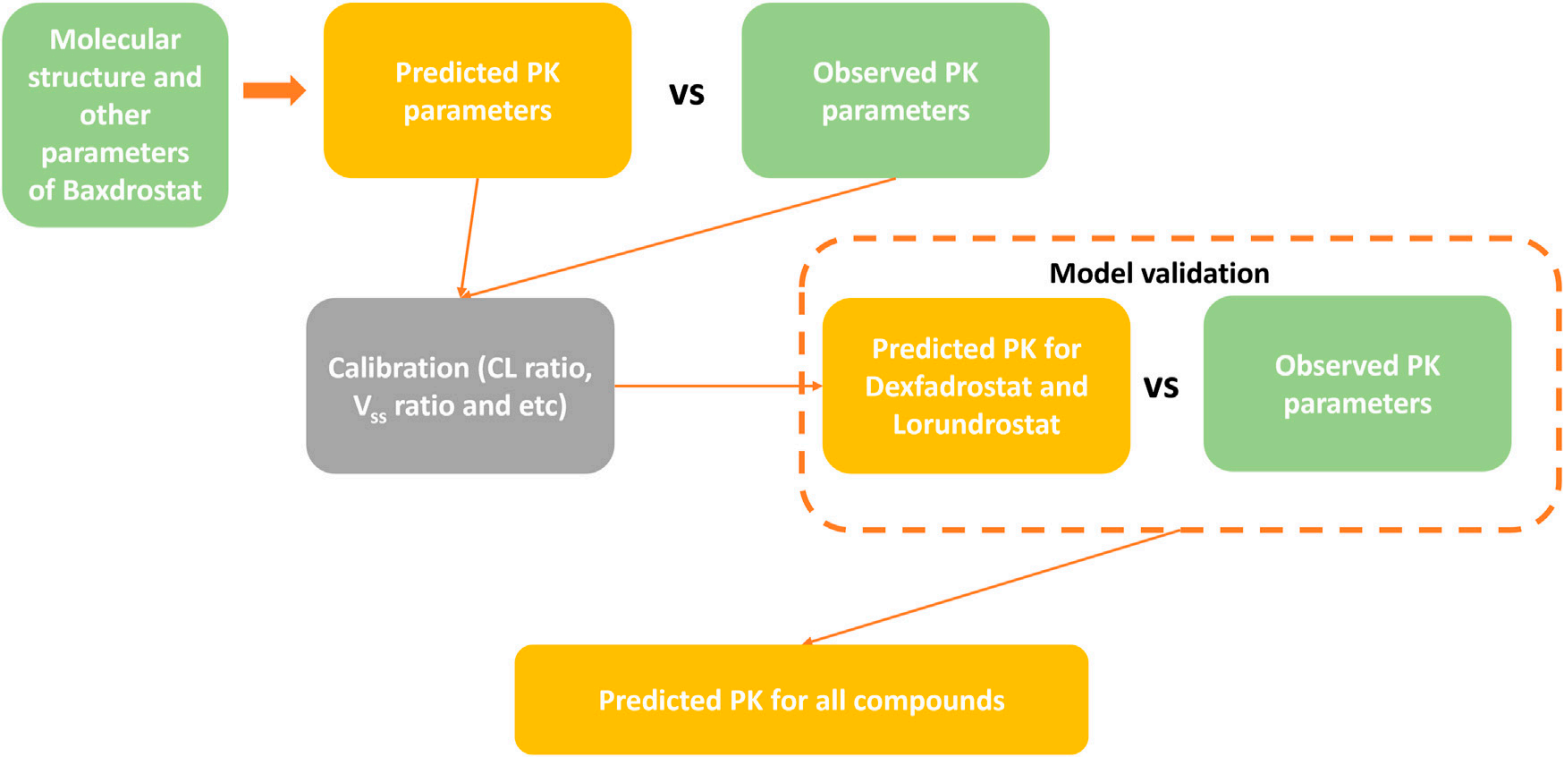
Workflow for predicting PK profiles of compounds using the AI-PBPK platform.

The workflow for predicting the PK profiles of a compound using the AI-PBPK model is shown in Figure 2. It consists of four steps: model construction, calibration, validation, and simulation. To identify a selective ASI with high potency, we selected Baxdrostat, an ASI currently with the most published research data, as the model drug. After constructing the ASI model, we predicted the PK parameters of Baxdrostat and compared the predictions with publicly available clinical trial data (Bogman et al., 2017; Freeman et al., 2023). The model was then calibrated by adjusting key parameters based on the comparison. Subsequently, we conducted external validation of the model using publicly available clinical PK data of two other ASIs, i.e., Dexfadrostat and Lorundrostat, with the next most publicly available clinical PK data. By comparing the predicted results with clinical data, we assessed the predictive performance of the AI-PBPK model for ASIs (Mulatero et al., 2023; Shimizu et al., 2024). Finally, the model was used to predict the PK profiles of all five compounds, i.e., Baxdrostat, BI689648, Dexfadrostat, Lorundrostat, and LCI699 (osilodrostat phosphate), an inhibitor of the 11β-hydroxylase. LCI699 was first approved as an orphan drug for the treatment of Cushing’s disease by the European Medicines Agency in 2020, and it also received FDA approval in the same year. In this study, it was used as a control (Papillon et al., 2015; Dormoy et al., 2023; Valentín-Goyco et al., 2023).

The model used to predict PD of the five compounds is an adaptation of Macdougall’s nonlinear model (Macdougall, 2006; Bogman et al., 2017), which is widely employed in dosage-response analyses. First, it is necessary to calculate the free plasma drug concentration based on the previously predicted plasma drug concentrations of all compounds, and then construct a PD model based on the free plasma drug concentration. The pharmacodynamic endpoint is mainly a compound’s inhibition of AS versus inhibition of 11β-hydroxylase. The ratio of a drug’s IC_50_ (half-maximal inhibitory concentration) toward 11β-hydroxylase to that toward AS is defined as its selectivity index (SI).

### 2.2 Literature search

Using PubMed, Google Scholar and ClinicalTrials.gov with keywords such as aldosterone synthase inhibitor, CYP11B2, and compound name (e.g., CIN-107 and MLS-101), we compiled the aforementioned five compounds, listed in Table 1. Their associated PK/PD data were either found in the collected literature or extracted from the official website of the investigating company (Shimizu et al., 2024). The SMILES codes and structural formulae for all five compounds were obtained from PubChem (https://pubchem.ncbi.nlm.nih.gov/).

**TABLE 1:** Essential data related to all five aldosterone synthase inhibitors.

Compound	SMILES code	Alias	Company	Highest R&D status	Target	Available data
Baxdrostat	CCC(=O)NC1CCCC2 = C1C = NC = C2C3 = CC4 = C(C=C3)N(C(=O)CC4)C	CIN-107, RO-6836191	CinCor, AstraZeneca PLC	Phase 3	Aldosterone synthase	PK; PD (Bogman et al., 2017; Freeman et al., 2023)
Dexfadrostat	C1CC(N2C = NC = C2C1)C3 = CC = C(C=C3)C#N	DP-13, (R)-Fadrozole (Damian Pharma), DP13	Damian	Phase 2	Aldosterone synthase	PK; PD (Rigel et al., 2010; Weldon et al., 2016; Mulatero et al., 2023)
Lorundrostat	CC1 = CC = C(C=C1)C2 = CN = NC(=N2)N3CCN(CC3)CC(=O)NC4CCC(CC4)NC(=O)C	MLS-101, MT 4129	Mineralys, Mitsubishi	Phase 3	Aldosterone synthase	PK; PD (Laffin et al., 2023; Mineralys Therapeutics, 2023; Shimizu et al., 2024)
BI-689648	COCC1 = CC(=CN = C1)C2 = CC3 = C(N=C2)N(CCC3)C (=O)N	—	Boehringer Ingelheim	—	Aldosterone synthase	PD (Weldon et al., 2016)
LCI-699	C1CC2 = CN = CN2C1C3 = C(C=C(C=C3)C#N)F	Osilodrostat, Osilodrostat phosphate (JAN/USAN), 5YL4IQ1078 (UNII code), LCI-699-NX, Isturisa, イスツリサ	Novartis, Recordati SpA et al.	Approved for listing	11β-hydroxylase	PD (Weldon et al., 2016)

Drug data updated as of September 2024.

### 2.3 Machine learning and PBPK/PD modeling

#### 2.3.1 Machine learning

Machine learning was used on the B^2^O Simulator^®^ platform to predict drug-specific parameters. Gilmer’s Message Passing Neural Networks (MPNNs) model (Gilmer et al., 2017), a subclass of graphical neural networks (GNNs) (Wu et al., 2021), is particularly useful for analyzing the chemical structure of a compound and predicting its pharmacological properties (Tang et al., 2023), such as fraction unbound in plasma (f_up_), blood to plasma ratio (bpr), steady-state volume of distribution (V_ss_), and gastrointestinal absorption constant (gi-ka) (Jiménez-Luna et al., 2021). The apparent clearance was also predicted using the M5P algorithm (Wang and Witten, 1997; Freitas et al., 2015). A detailed discussion of the performance of the underlying ML model and the use of the model to generate key compound parameters can be found in a previous article using the platform (Wu et al., 2024).

#### 2.3.2 Data sources for machine learning

Five ADME parameters, namely, f_up_ (f_up_ = 1-ppbr; ppbr, plasma protein binding ratio), CL_app_ (CL_app_ = V_ss_*0.693/half-life), V_ss_ (V_ss__per kg), gi_ka (gi_ka = 2*P_eff_/radius of the small intestine; Log (P_eff_) = 0.6795Log (P_app_)-0.3355), and bpr, were modeled by an ML approach. The diameter of the small intestine is 2.5 cm (Helander and Fändriks, 2014), thus its radius is 1.25 cm. Data for ppbr, V_ss_, half-life and P_app_ were obtained from the Therapeutics Data Commons (TDC) database. The Python package ‘PYTDC’ was installed and the corresponding dataset was used in this package. The bpr data were obtained from Mamada’s work (Mamada et al., 2021). The five datasets consist of two vectors of molecular structure shown by the SMILES code and the corresponding ADME parameters for each molecule. Most of the data sources for ML come from the TDC database (datasets: ppbr, TDC. PPBR_AZ, size: 1797; V_ss_, TDC. VD_ss__Lombardo, size: 1,130; half-life, TDC. Half_Life_Obach, size: 667; P_app_, TDC. Caco2_Wang, size: 906; bpr, Mamada’s work, size: 461) (Huang et al., 2021; Mamada et al., 2021).

#### 2.3.3 PBPK/PD modeling

In the study, the whole-body PBPK model (Wendling et al., 2015; Wendling et al., 2016) was used, which consists of 14 tissue compartments, including lungs, heart, brain, muscle, fat, skin, spleen, pancreas, liver, stomach, intestine, bone, kidney and other parts of the body, and two blood compartments (arterial and mixed venous). Assuming equilibrium of drug distribution in tissues and plasma, the degree of distribution was characterized by the blood partition coefficient (K_p_). The rate equation for the tissue compartment is as follows (Equation 1):

$$\frac{{dA}_{T}}{dt}=\frac{Q_{T}}{V_{VEN/ART}}\cdot A_{VEN/ART}-\frac{Q_{T}}{V_{T}K_{p}}\cdot A_{T}$$ (1)

Where $A_{T}$ denotes the drug amount (mg), $V_{T}$ is the volume (L), and $Q_{T}$ is the blood flow (L/h) for the 14 different tissues. ${A\;}_{VEN/ART}$ and $V_{VEN/ART}$ are the amount (mg) and volume (L), respectively, of either mixed venous blood (for the lungs) or arterial blood (for tissues other than the lungs).

Additionally, the PD model describes the correlation between the plasma free drug concentration (plasma drug concentration *f_up_) and the inhibition of AS and 11β-hydroxylase. The *in vitro* inhibition assay evaluated the inhibitory effect of an ASI on the activities of the two enzymes by measuring the enzymatic conversion of 11-deoxycortisol to cortisol for CYP11B1, and 11-deoxycorticosterone (11-DOC) to aldosterone for CYP11B2, and was presented as the inhibition constants of both enzymes by the free base of the ASI (Shimizu et al., 2024). Thus, based on the free plasma drug concentration of the ASI and the corresponding inhibition constants of the compound on both enzymes, i.e., the IC_50_ in this study (Table 6), PD modeling was performed separately for each enzyme. The Exposure-Response (ER) relationship is as follows (Equation 2):

$$E=E_{0}-\frac{I_{\max}*C}{C+{IC}_{50}}$$ (2)

Where C is the plasma concentration, $E_{0}$ is the percentage change from baseline in plasma aldosterone concentration at C = 0 (theoretically 0%), and $I_{\max}$ is the maximal inhibitory effect, which is typically 100% and indicates complete inhibition.

#### 2.3.4 Design of simulation studies

Once the required parameters of Baxdrostat were predicted with the ML model, the constructed PBPK model was used to simulate the drug’s PK profiles. The model was then calibrated appropriately based on the differences between the simulated results and the observed data. Following calibration, Lorundrostat and Dexfadrostat were selected to validate the model, and the fitting effect was evaluated by comparing the simulated outcomes with observed values. Upon validation, the single ascending dose (SAD) and multiple ascending dose (MAD) PK simulation of all drugs at dosages of 0.5 mg, 1 mg, 3 mg, 10 mg and 30 mg was selected, and the corresponding PD was simulated based on the obtained PK parameters. The dosing interval for all multiple doses was 24 h. Due to the high dosage of Lorundrostat in the clinical trials, the existing dosages of 5 mg, 10 mg, 20 mg, 50 mg and 100 mg in the clinical trials were used for SAD simulation, and dosages of 3 mg, 12.5 mg, 50 mg, and 100 mg were selected for MAD simulation. The PK and PD properties of the compounds at different dosages were analyzed and compared to determine the optimal combination of PK, PD, and dosage for the candidate drugs.

### 2.4 Software

The PBPK model was built, fitted and used for prediction using the web-based B^2^O Simulator^®^ at https://simulation.b2osim.cn/signin. To access the AI-PBPK module, users must register and log in, and then input the chemical structure to predict a drug’s PK parameters. The maximum serum concentration reached by a drug in the body and the area under the curve are calculated within a confidence interval (2.5%–97.5%). When the predicted data are less than 1/2 or more than twice of the observations from clinical studies, the deviation is considered significant. All data and graphs were generated using Microsoft^®^ Excel 2021 (64-bit) and R version 4.3.0 software.

## 3 Results

### 3.1 Simulation, calibration, and validation of PK

#### 3.1.1 Simulation of Baxdrostat’s PK

**FIGURE 3:**
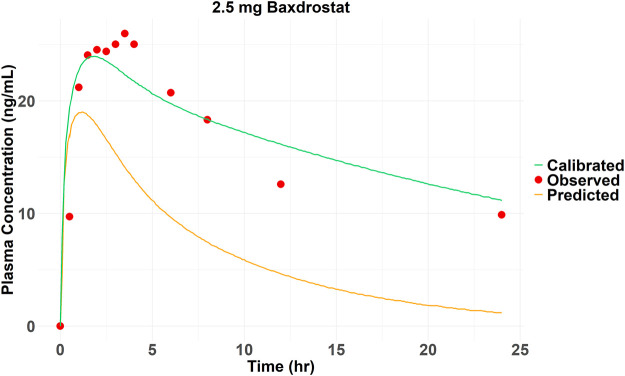
Observed and predicted (before and after calibration) plasma drug concentrations of 2.5 mg Baxdrostat over time.

The SMILES code for Baxdrostat (CCC(=O)NC1CCCC2 = C1C = NC = C2C3 = CC4 = C(C=C3)N(C(=O)CC4)C), together with the codes of the other four drugs (Table 1), were entered into the AI-PBPK platform to generate ADME and physicochemical parameters shown in Table 2. Baxdrostat’s f_up_ was from Bogman’s work (Bogman et al., 2017). The simulated PK parameters of a single dose of 2.5 mg (same as the clinical trial dosage) of Baxdrostat and the observed data (Freeman et al., 2023) are shown in Table 3. The simulated drug exposure values, which represent the relationship between plasma drug concentration and time, are lower than the observed mean exposure values. Drug exposure is primarily reflected through indicators such as the area under the concentration (AUC)-time curve, maximum plasma concentration (C_max_), and time to reach maximum concentration (T_max_). The simulated PK profiles and observations for a 2.5 mg single dose of Baxdrostat are shown in Figure 3. The observed and predicted Mean Absolute Error (MAE), Mean Squared Error (MSE), Root Mean Square Error (RMSE) and R-Square (*R* ^2^) values are 7.81, 73.0, 8.54 and 0.54, respectively. Since the simulated results deviated significantly from the observed data, the model was calibrated.

**TABLE 2:** ADME parameters of five ASIs predicted by ML before calibration.

Symbol	Parameters (Unit)	Baxdrostat	BI689648	Lorundrostat	Dexfadrostat	LCI699
MW	molecular weight (g/mol)	363.50	298.36	451.6	223.27	227.24
f_up_	unbound fraction to plasma protein	0.26^a^	0.18	0.11	0.18	0.20
bpr	blood-to-plasma ratio	0.96	1.04	0.66	0.74	0.77
gi_ka	GI absorption rate constant (h^-1^)	0.96	0.73	0.36	0.86	1.63
CL_app_	clearance (L/h)	14.83	14.32	39.36	71.11	95.05
V_ss__perKg	V_ss_ (L/kg)	1.37	1.29	1.54	1.67	1.58
kp_bone	bone: plasma	2.55	0.24	0.33	1.21	0.73
kp_brain	brain: plasma	45.07	0.22	0.29	1.03	0.63
kp_adipose	adipose: plasma	18.05	1.29	2.20	11.88	6.58
kp_heart	heart: plasma	7.31	0.37	0.46	1.55	0.97
kp_kidney	kidney: plasma	7.12	0.28	0.30	0.81	0.55
kp_gut	gut: plasma	9.81	0.30	0.36	0.98	0.64
kp_liver	liver: plasma	4.75	0.23	0.26	0.76	0.50
kp_lung	Lung: plasma	9.41	0.26	0.30	0.31	0.29
kp_muscle	muscle: plasma	8.02	0.13	0.19	0.34	0.25
kp_skin	skin: plasma	37.69	0.39	0.46	1.05	0.73
kp_spleen	spleen: plasma	5.39	0.19	0.22	0.41	0.31
Kp_scaler	Kp scaler	0.16	3.75	3.33	0.91	1.46

^a^ The f_up_ of Baxdrostat is from the literature.

**TABLE 3:** Important PK parameters of 2.5 mg Baxdrostat simulated before and after calibration, and observed ones.

PK parameters	Simulated before calibration	Observed^a^	Simulated after calibration
AUC_0-24_ (ng*hr/mL)	157.25	365.79	396.55
C_max_ (ng/mL)	18.99	28.09	23.97
T_1/2_ (hr)	6.10	28.37	21.80
T_max_ (hr)	1.20	3.00	1.80

^a^The observed data are from the literature (Freeman et al., 2023).

#### 3.1.2 Calibration of Baxdrostat’s PK

The Kp_scaler is a scaling factor applied to modify the Kp values, which represent the tissue/blood partition coefficient. The Kp_scaler calibrates the predicted Kp values to better fit experimental data or improve the model’s accuracy. The size of Kp_scaler varies based on the predicted V_ss__per Kg. The simulated AUC_0-24_ (157.25 ng*hr/mL) of Baxdrostat was smaller than the observed value (365.79 ng*hr/mL), suggesting that the model overestimated the distribution of the drug in the tissue, resulting in lower simulated blood drug concentrations than the observations. Therefore, by adjusting the Kp_scaler to an extent, the drug distributed into the tissues was reduced, thereby bringing the simulated drug concentration in the blood to be closer to the observed value. The simulated plasma elimination half-life (≈6 h) was shorter than the actual one (28.37 h). The drug’s predicted clearance (CL_app_ = 14.83 L/h) was higher than the observed value (2.5 mg Baxdrostat, CL_app_ = 3.28 L/h), suggesting that the actual drug clearance is faster than predicted. By reducing the predicted CL_app_, the biological half-life was prolonged, bringing the modeled values closer to the observed ones. The final calibration for parameters was Kp_scaler * 0.85 and CL_app_/4.7. The PK curve for Baxdrostat after calibration is shown in Figure 3, and it can be seen that the calibrated curve is closer to the observed one. The observed and predicted (after calibration) MAE, MSE, RMSE and *R* ^2^ values are 2.13, 10.87, 3.30 and 0.83, respectively. These two calibration coefficients were also applied to the other compounds. The drugs’ parameters after calibration are shown in Table 4. The PK parameters generated after Baxdrostat calibration can also be seen in Table 3. The AUC_0-24_ and C_max_ are closer to the observed values after calibration.

**TABLE 4:** CL_app_ and Kp_scaler of five ASIs after calibration.

Symbol	Baxdrostat	BI689648	Lorundrostat	Dexfadrostat	LCI699
CL_app_	3.15	3.05	8.37	15.13	20.22
Kp_scaler	0.14	3.19	2.83	0.78	1.24

#### 3.1.3 Model validation with Lorundrostat and Dexfadrostat

**FIGURE 4:**
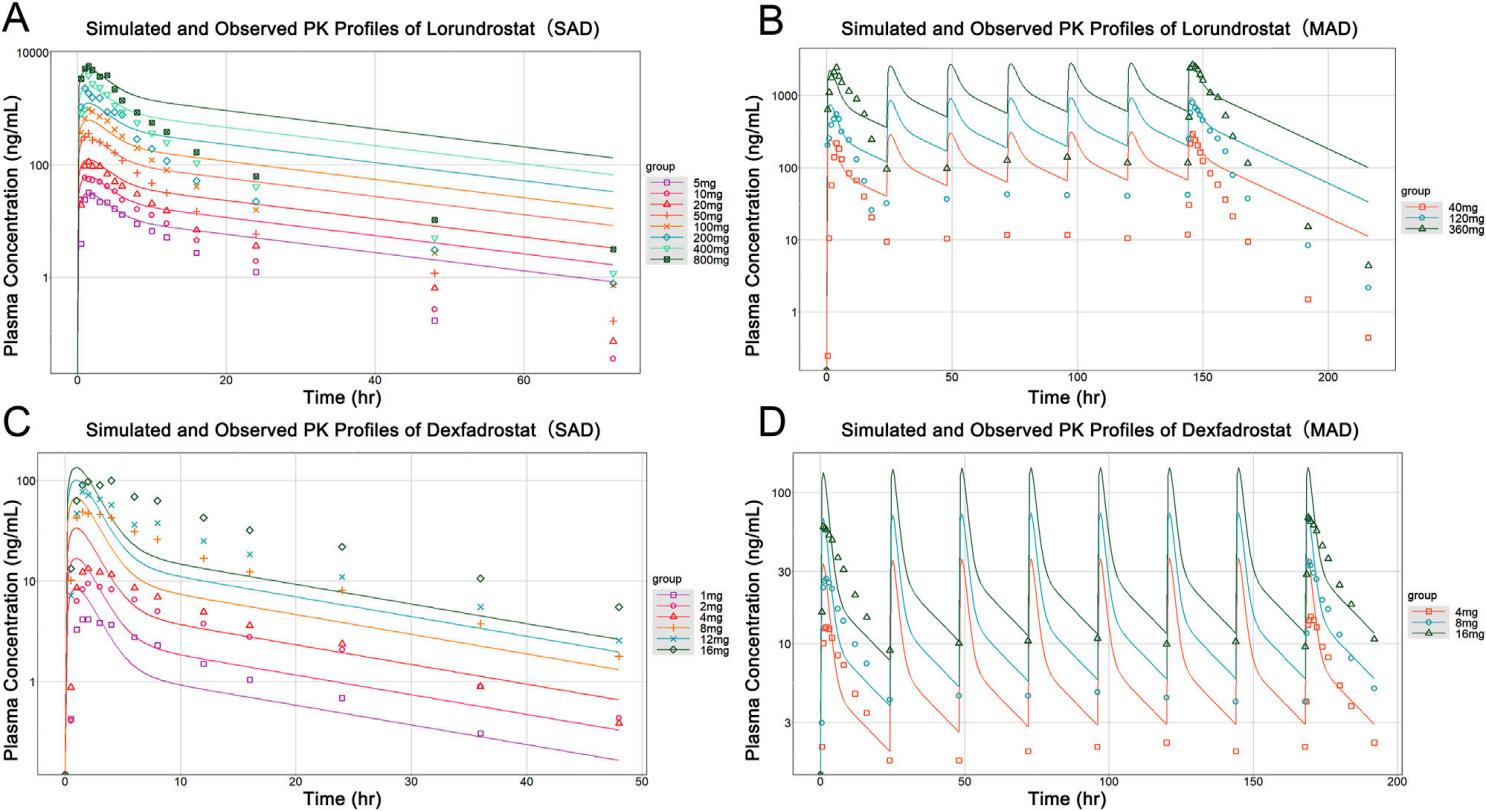
Observed versus predicted PK profiles of Lorundrostat and Dexfadrostat at different dosages. **(A)** Simulated and observed profiles for a single dose of Lorundrostat (5, 10, 20, 50, 100, 200, 400, 800 mg). **(B)** Simulated and observed profiles of Lorundrostat at multiple doses (40 mg, 120 mg, 360 mg). **(C)** Simulated and observed profiles for a single dose of Dexfadrostat (1 mg, 2 mg, 4 mg, 8 mg, 12 mg, 16 mg). **(D)** Simulated and observed profiles of Dexfadrostat at multiple doses (4 mg, 8 mg, 16 mg).

**FIGURE 5:**
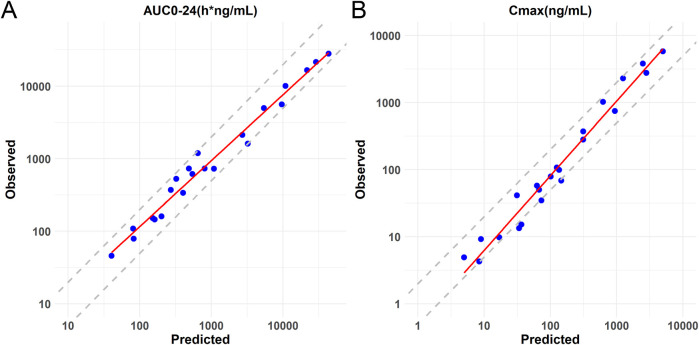
Logarithmic coordinate plots of predicted versus observed values of AUC_0-24_ and C_max_ for Baxdrostat, Dexfadrostat and Lorundrostat. The solid line represents the linear regression fit of the data. **(A)** Predicted versus observed AUC_0-24_ with 2-fold and 0.5-fold deviation lines. **(B)** Predicted versus observed C_max_ with 2-fold and 0.5-fold deviation lines.

After calibration, the predictive ability of the model was further validated by testing Lorundrostat and Dexfadrostat on it. Figure 4 shows the predicted and observed PK results for Lorundrostat and Dexfadrostat as semi-logarithmic curves. The hollow markers in the figure are observations and the solid lines are predictions. The predicted plasma concentrations of the drugs Lorundrostat and Dexfadrostat for validation were consistent with the concentration profiles observed in the literature at the initial stage after the first dose. But the plasma concentrations at the elimination stage of Lorundrostat were high and the rates of elimination slowed for both single and multiple doses, while the plasma concentrations of Dexfadrostat at the elimination stage was low for single and multiple doses and the elimination rates was fast. However, most of the ratios of predicted-to-observed AUC_0-24_, C_max_, and T_max_ for single-dose and multiple-dose administration were within 1/2 to 2-fold (Table 5), except the C_max_ of Dexfadrostat at 4 mg for SAD, and 4 mg, 8 mg, and 16 mg for MAD were not within the 2-fold error range. The coefficient of determination (*R* ^2^) of the predicted and observed values of AUC_0-24_ and C_max_ was 0.986 and 0.960, respectively (Figure 5). The simulation was based on a single virtual healthy subject. The results of the analyses showed that the model reasonably predicted drug exposure within a certain error range and is of great value for candidate screening.

**TABLE 5:** Ratio of predicted to observed C_max_, T_max_, AUC_0-24_ values for three ASIs at different dosages and dosing modules.

Name	Dosage (mg)	Dosing module	Ratio		
			RC_max_	RT_max_	RAUC_0-24_
Baxdrostat	0.5	SAD	1.01	0.90	1.04
Baxdrostat	0.5	MAD	0.97	0.50	1.00
Dexfadrostat	1	SAD	1.98	0.68	0.88
Dexfadrostat	2	SAD	1.73	0.68	0.74
Dexfadrostat	4	SAD	2.54	0.69	1.11
Dexfadrostat	8	SAD	1.35	1.09	0.61
Dexfadrostat	12	SAD	1.28	1.09	0.66
Dexfadrostat	16	SAD	1.37	0.68	0.54
Dexfadrostat	4	MAD	2.40	0.50	1.26
Dexfadrostat	8	MAD	2.11	0.73	1.20
Dexfadrostat	16	MAD	2.14	1.31	1.11
Lorundrostat	5	SAD	0.76	0.93	0.73
Lorundrostat	10	SAD	1.08	0.93	0.88
Lorundrostat	20	SAD	1.17	0.93	1.50
Lorundrostat	50	SAD	0.84	0.94	1.28
Lorundrostat	100	SAD	0.61	0.94	1.09
Lorundrostat	200	SAD	0.54	1.40	1.08
Lorundrostat	400	SAD	0.66	1.40	1.31
Lorundrostat	800	SAD	0.86	0.94	1.55
Lorundrostat	40	MAD	1.12	0.77	2.00
Lorundrostat	120	MAD	1.26	1.07	1.72
Lorundrostat	360	MAD	1.02	1.10	1.35

### 3.2 Simulation of PK for all five compounds

**FIGURE 6:**
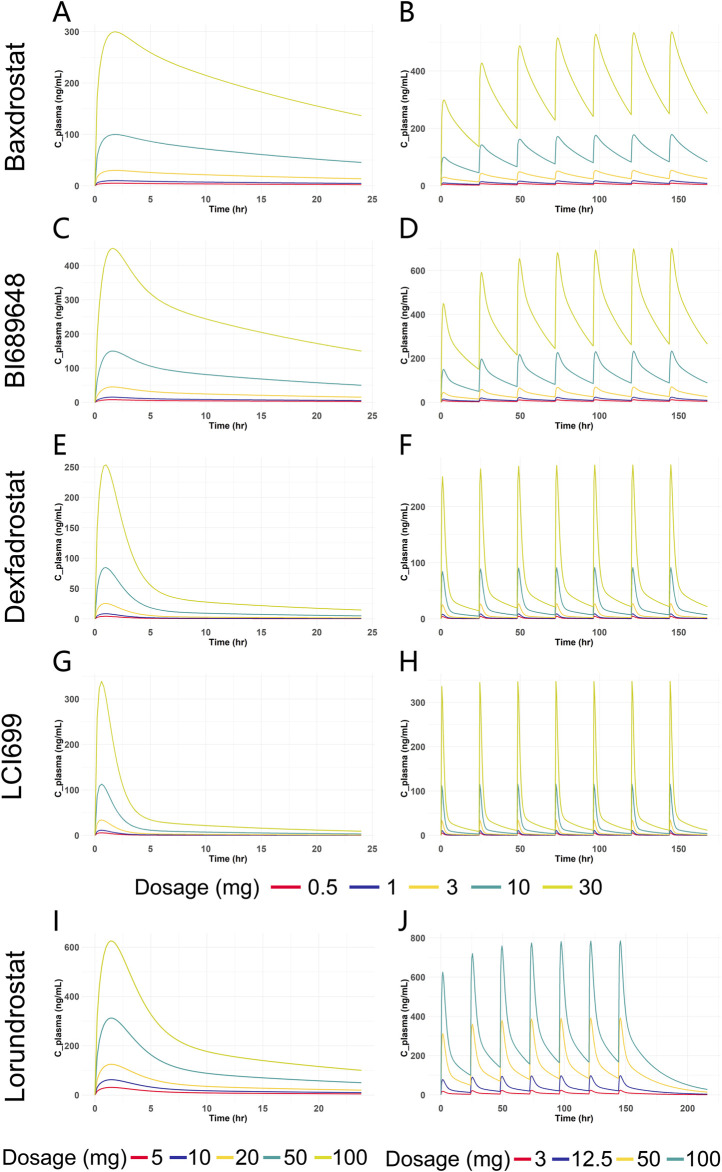
Predicted plasma drug concentration versus time curves for five ASIs at different dosages. **(A)** Blood drug concentration-time curves of Baxdrostat at SAD. **(B)** Blood drug concentration-time curves of Baxdrostat at MAD. **(C)** Blood drug concentration-time curves of BI689648 at SAD. **(D)** Blood drug concentration-time curves of BI689648 at MAD. **(E)** Blood drug concentration-time curves of Dexfadrostat at SAD. **(F)** Blood drug concentration-time curves of Dexfadrostat at MAD. **(G)** Blood drug concentration-time curves of LCI699 at SAD. **(H)** Blood drug concentration-time curves of LCI699 at MAD. **(I)** Blood drug concentration-time curves of Lorundrostat at SAD. **(J)** Blood drug concentration-time curves of Lorundrostat at MAD.

After model validation, dosages of 0.5 mg, 1 mg, 3 mg, 10 mg, and 30 mg were selected for SAD and MAD PK simulation for Baxdrostat, BI689648, Dexfadrostat, and LCI699. Due to the large dosage of Lorundrostat in the clinical trials, single dose of 5 mg, 10 mg, 20 mg, 50 mg and 100 mg, and multiple doses of 3 mg, 12.5 mg, 50 mg and 100 mg were selected for PK simulation. The PK simulation results of all the compounds are shown in Figure 6. The plasma drug concentration profiles of all the compounds increased in a dosage-dependent manner as shown by the plasma drug concentration graphs for single and multiple doses. The predicted AUC_0-24_ and C_max_ values for all the compounds are provided in Supplementary Table S1, which shows that under both single and multiple doses conditions, the AUC_0-24_ for Baxdrostat, BI689648, and Dexfadrostat is higher than that of LCI699 at equivalent doses; while the C_max_ of LCI699 is slightly higher than that of Dexfadrostat at the same dosage, its rapid elimination resulted in a comparatively lower AUC_0-24_ than that of Baxdrostat, BI689648, and Dexfadrostat at the same dosage. Lorundrostat has the lowest C_max_ at a single dose of 10 mg, but its AUC_0-24_ is higher than that of both Dexfadrostat and LCI699 at the same dosage, and the drug is eliminated slowly enough to achieve a certain blood concentration at steady state.

### 3.3 Simulation of PD for all five compounds

**FIGURE 7:**
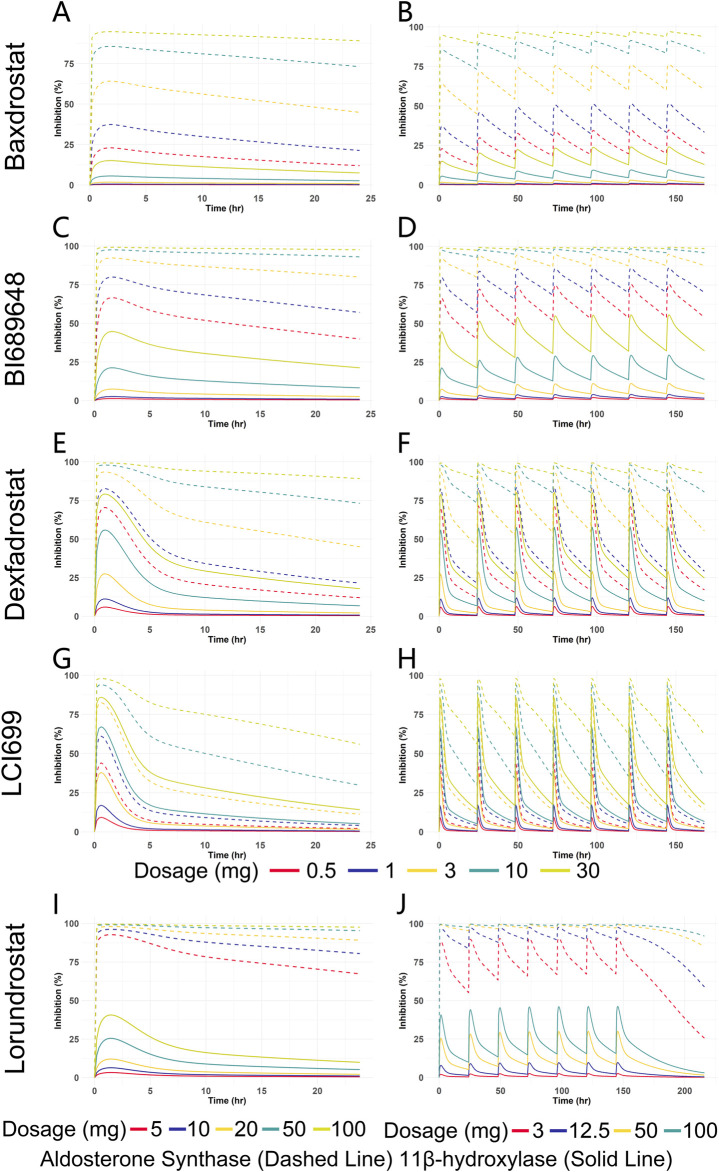
Predicted inhibition rate versus time curves for five ASIs at different dosages. **(A)** Enzyme inhibition over time for Baxdrostat at SAD. **(B)** Enzyme inhibition over time for Baxdrostat at MAD. **(C)** Enzyme inhibition over time for BI689648 at SAD. **(D)** Enzyme inhibition over time for BI689648 at MAD. **(E)** Enzyme inhibition over time for Dexfadrostat at SAD. **(F)** Enzyme inhibition over time for Dexfadrostat at MAD. **(G)** Enzyme inhibition over time for LCI699 at SAD. **(H)** Enzyme inhibition over time for LCI699 at MAD. **(I)** Enzyme inhibition over time for Lorundrostat at SAD. **(J)** Enzyme inhibition over time for Lorundrostat at MAD.

Clinical PD endpoints for ASIs are not limited to the change in seated mean systolic blood pressure from baseline to 8 weeks of treatment. For model construction, with limited *in vitro* clinical trial data and inconsistent PD endpoint metrics, the choice of IC_50_ as an input to the PD model allows for a direct comparison of the inhibitory effects of different compounds on AS and 11β-hydroxylase. Therefore, selective inhibition of AS without affecting 11β-hydroxylase is an observable indicator for PD evaluation in our study. By comparing the trend of the inhibition rate of a compound on AS and 11β-hydroxylase with dosage change, we can obtain the dosage range of the compound reaching the optimal inhibition rate and can compare different compounds at the same dosage or with the same inhibition effect. This analysis is limited for not fully reflecting the PD endpoints of clinical trials, but it can provide ideas for dosage recommendation and comparison of the efficacy of different compounds. The E_max_ model was used to predict the PD of all five compounds based on plasma free drug concentrations, and the IC_50_ was used as an input parameter (Table 6). For Baxdrostat, the IC_50_ is from the clinical data (Bogman et al., 2017). For the other drugs, the IC_50_ data are from monkey adrenal gland homogenate (Weldon et al., 2016; Laffin et al., 2023). The enzyme inhibition rates of single and multiple doses predicted for all the compounds are shown in Figure 7.

**TABLE 6:** IC_50_ and selective index of five ASIs for 11β-hydroxylase and aldosterone synthase for PD prediction.

Name	IC_50_ (nmol/L) and SI				
	LCI699 (Weldon et al., 2016)	Dexfadrostat (Weldon et al., 2016)	BI 689648 (Weldon et al., 2016)	Baxdrostat (Bogman et al., 2017)	Lorundrostat (Laffin et al., 2023; Mineralys Therapeutics, 2023; Shimizu et al., 2024)
11β-hydroxylase	77	94	310	1,310	475
Aldosterone synthase	10	2.5	2.1	13	1.27
SI^a^	7.7	38	149	100	374

^a^ SI, is defined as the ratio of IC_50_ for 11β-hydroxylase over IC_50_ for aldosterone synthase.

It can be seen that the inhibition of the enzymes increased in a dosage-dependent manner. As can be seen from the multiple doses plot, when the steady state was reached, Baxdrostat at a dosage of 3 mg showed a 65% inhibition of AS. When the dosage was 10 mg, the inhibition rate of AS reached 85%, the inhibition of 11β-hydroxylase began to increase significantly, and the selectivity of the compound gradually decreased. Therefore, the optimal dosage of Baxdrostat predicted in this study is 10 mg, with an AS inhibition rate of 85%. In the clinical trials, after the healthy subjects received a single increasing dosage of Baxdrostat, the plasma aldosterone decreased in a dosage-dependent manner with the maximum effect reached at 10 mg (Bogman et al., 2017). The predicted best inhibitory effect of Baxdrostat was also reached at 10 mg, with a strong inhibition of AS and weak inhibition of 11β-hydroxylase, echoing the clinical trial data. When BI689648 was administered at a dosage of 3 mg, the inhibition of AS reached 90%, at which point the inhibition of 11β-hydroxylase began to increase and the side effects started to become apparent. Therefore, the optimal dosage of BI689648 is 3 mg, achieving an AS inhibition rate of 90%. At a dosage of 3 mg, the inhibitory effect of Baxdrostat on AS was only 65%, which was lower than that of BI689648 at the same dosage. When Lorundrost was administered at a dosage of 3 mg, the inhibition of AS reached 75%, which was slightly lower than that of BI689648 at 3 mg, and was comparable to the effect of BI689648 at 1 mg. As the dosage increased to 12.5 mg, the inhibition of AS reached 90%, at that point the inhibition of 11β-hydroxylase began to increase. The inhibition at this point was comparable to that of BI689648 at 3 mg. Therefore, the optimal dosage of Lorundrost in our predicted PD is 12.5 mg, and the inhibition rate of AS can reach 90%. With dosage increasing, the inhibition of 11β-hydroxylase by Baxdrostat, Lorundrost, and BI689648 becomes more pronounced. For Dexfadrostat and LCI699, the inhibition of AS and 11β-hydroxylase are very close to each other, thus neither drug is very selective.

## 4 Discussion

In this study, we used all available data of Baxdrostat to build the best possible general AI-PBPK model for ASIs and used clinical PK data for correction. Data from clinical PK trials of Dexfadrostat and Lorundrost were used in subsequent external validation to assess the model’s performance and generalizability across clusters. This validation process allowed for an assessment of the model’s ability to simulate biological processes, identify biases, and refine its predictive capabilities, ensuring credibility and reliability for real-world applications (Collins et al., 2024). The validation component is therefore of great value in improving the goodness of fit of the model. The results of model validation showed that the ratios of most predicted-to-observed AUC_0-24_ and C_max_ values were in the range of 1/2-fold–2-fold, reflecting the validity of the model in simulating PK behaviors. Yet, four C_max_ ratio data fell outside of this range, suggesting deficiencies in parameter estimation. And in the absence of sufficient clinical trial data to calibrate the model, the predictions may be biased due to the lack of calibration conditions (Huang et al., 2002).

PK prediction of all five compounds at the same dosage showed that Baxdrostat, BI689648, Lorundrostat had higher AUC_0-24_ than LCI699, and the highest drug exposure was found for BI689648, followed by Baxdrostat, Dexfadrostat, and LCI699. The AUC_0-24_ of Lorundrostat was intermediate between that of Baxdrostat and Dexfadrostat for a single dose of 10 mg and multiple doses of 3 mg. The PD results corresponding to PK showed that when the inhibition rate of 11β-hydroxylase did not start to fluctuate, the compound with the highest selectivity for AS and 11β-hydroxylase was Lorundrostat at 3 mg, followed by BI689648 at 1 mg, and Baxdrostat at 3 mg, and their inhibition of AS could reach 80%, 75% and 65%, respectively. This trend was consistent with the *in vitro* observational data (Lorundrostat SI: 374, BI689648 SI: 149, Baxdrostat SI: 100, Dexfadrostat SI: 38) (Weldon et al., 2016; Bogman et al., 2017; Laffin et al., 2023; Shimizu et al., 2024). LCI699 at a dosage of 0.5 mg exhibited a potent inhibition of 11β-hydroxylase activity, further substantiating the efficacy of LCI699 as a 11β-hydroxylase inhibitor (LCI699 SI: 7.7) (Weldon et al., 2016). The predicted PD trend matched the clinically observed one, validating the capacity of the model in translational research. Thus, PD prediction for more similar ASIs based on their structural formulae could be performed to assess their potential (Mehić, 2011). The pivotal Phase III clinical trial of Lorundrostat (Launch-HTN, NCT06153693) successfully met its primary endpoint, resulting in a 16.9 mmHg reduction in systolic blood pressure after treatment with Lorundrostat at 50 mg, in contrast to a placebo-adjusted reduction of 9.1 mmHg (p < 0.0001), as shown by automated office blood pressure measurement at week 6. Additionally, the trial met a predefined endpoint at the end of treatment (week 12), where Lorundrostat at 50 mg led to a 19.0 mmHg reduction in systolic blood pressure, against an 11.7 mmHg placebo-adjusted reduction (p < 0.0001). This is of great significance for the treatment of Uncontrolled or Resistant Hypertension with ASIs. It demonstrates to some extent the feasibility of our PD prediction. Thus, in the preclinical stage, PD prediction based only on limited data and compounds structural formulae can provide ideas for dosage recommendations and efficacy comparisons of different compounds, and such predictive models are of great value.

Nevertheless, inconsistence existed between certain predicted and observed parameters. Results of a randomized, placebo-controlled, dosage-varied trial in adults with uncontrolled hypertension taking two or more antihypertensive medicines (Laffin et al., 2023) showed that Lorundrostat resulted in a reduction in serum aldosterone at all dosages, and there were only small increases in serum cortisol. In our predicted PD, the inhibition rate of 11β-hydroxylase became apparent for Lorundrostat at 12.5 mg, which showed some differences from the results in human clinical trials. This may be due to that the gene expression of CYP11B2 and CYP11B1 in the adrenal gland is regulated by epigenetic modification, which contributes to autonomous aldosterone and cortisol synthesis (Takeda et al., 2023). In addition, the simulated inhibition rates did not take into account the effect of differences in drug metabolism *in vivo*, which involves highly complex cooperation between drug transporter proteins and drug-conjugating and metabolizing enzymes, as well as targeted programs of gene activation and proteasomal degradation pathways. Moreover, drug transport and metabolism in the intestine and liver mediate the systemic delivery of therapeutic compounds (Staudinger, 2013). In addition, using IC_50_ from different species as inputs to the E_max_ model and drawing conclusions based on predicted PD may be flawed and error-prone for predicting clinical effectiveness. Cross-species and in vitro-in vivo extrapolations require consideration of species-specific physiology, plasma protein binding, enzyme and transport kinetics, and tissue-specific gene expression profiles. Comprehensive considerations can increase the accuracy of cross-species and in vitro-in vivo extrapolations (Thiel et al., 2015).

Overall, for simulation of an ASI’s PD with the AI-PBPK model, the bias in results predicted by the model arises primarily from two factors: (1) the inherent imprecision of machine learning in predicting compound-specific parameters, influenced by the data sources and prediction methods employed, and (2) the combination of ML prediction bias with PBPK model bias, which amplifies inaccuracies in PK data and subsequent PD modeling. These biases are inherent limitations of current models, which hinder the models from fully accounting for variations across individuals, races, and disease populations (Cross et al., 2024). In 2024, Chen et al. systematically analyzed the main factors affecting prediction accuracy through a model-driven pharmacogenetic-pharmacodynamic (PG/PD) exploration with a machine learning approach (Chen et al., 2024). Approaches proposed by them to reduce bias include sensitivity analyses to identify key parameters and improve the relevance of data collection; the use of large-scale biomarker data to improve PK/PD associations; and the use of multiple machine-learning algorithms for cross-validation to improve the model’s robustness (Chen et al., 2024).

From the perspective of model generalizability, the AI-PBPK model can predict drug-specific parameters and PK exposure based solely on a compound’s molecular structure. In a previous work on proton pump inhibitors, the model validation showed a Pearson correlation coefficient (r) between 0.84 and 0.89, with consistent trends between predicted and observed PK data (Wu et al., 2024). In the current case of ASIs, the model’s fit to AUC_0-24_ and C_max_ was evaluated, both showing a coefficient of determination greater than 0.95. From the existing work on ASIs, there is considerable room for improvement in predicting certain drug-specific parameters, such as clearance. Overall, this study provides a method for predicting a compound’s PK/PD based on its structural formula at the drug discovery stage and presents an AI-PBPK/PD model for ASIs, which can predict the PK characteristics of similar ASIs in the human body, analyze the dose-exposure and exposure-effect, *etc.*, providing a reference for subsequent compound screening and clinical research.

## 5 Conclusion

The AI-PBPK model was employed to predict the PK profiles of five aldosterone synthase inhibitors, and the inhibition of aldosterone synthase and 11β-hydroxylase by the five compounds was predicted using a PD model based on their free blood concentrations. It is realized that a compound’s PK/PD in the human body can be obtained through predictions based on its structural formula at the drug discovery stage. This can accelerate ASIs screening, avoiding the extensive trial and error process. Yet, there is still considerable room for improvement in model fitting. We will carry out more case studies to further prove the model’s effectiveness and continuously optimize the model to improve its overall prediction accuracy. With further improvement, our AI-PBPK model on the B^2^O simulation platform shall have great potential to predict the therapeutic effects of drug candidates at the early stages of drug discovery.

## Data Availability

The original contributions presented in the study are included in the article/Supplementary Material, further inquiries can be directed to the corresponding author.
