# Supplementary material for: Prediction of pharmacokinetic/pharmacodynamic properties of aldosterone synthase inhibitors at drug discovery stage using an artificial intelligence-physiologically based pharmacokinetic model
Source: Front Pharmacol. 2025 Apr 28;16:1578117. doi: 10.3389/fphar.2025.1578117 (PMC12066422; doi:10.3389/fphar.2025.1578117)
Supplement: Supplementary file 1 [file Table1.docx]

Supplementary Material

# Supplementary Tables

Table 1 Predicted C_max_, AUC_0-24_ for all compounds at SAD and MAD administration.

| Name | Dosage (mg) | Dosing Module | C_max_ (ng/mL) | AUC_0-24_ (hr*ng/mL) |
| --- | --- | --- | --- | --- |
| Baxdrostat | 0.5 | SAD | 4.99 | 82.31 |
| Baxdrostat | 1 | SAD | 9.99 | 164.62 |
| Baxdrostat | 3 | SAD | 29.96 | 493.86 |
| Baxdrostat | 10 | SAD | 99.86 | 1646.20 |
| Baxdrostat | 30 | SAD | 299.57 | 4938.61 |
| Baxdrostat | 0.5 | MAD | 8.94 | 151.71 |
| Baxdrostat | 1 | MAD | 17.89 | 303.41 |
| Baxdrostat | 3 | MAD | 53.66 | 910.24 |
| Baxdrostat | 10 | MAD | 178.86 | 3034.12 |
| Baxdrostat | 30 | MAD | 536.59 | 9102.36 |
| BI689648 | 0.5 | SAD | 7.51 | 98.82 |
| BI689648 | 1 | SAD | 15.01 | 197.65 |
| BI689648 | 3 | SAD | 45.04 | 592.95 |
| BI689648 | 10 | SAD | 150.12 | 1976.49 |
| BI689648 | 30 | SAD | 450.36 | 5929.48 |
| BI689648 | 0.5 | MAD | 11.68 | 170.41 |
| BI689648 | 1 | MAD | 23.36 | 340.81 |
| BI689648 | 3 | MAD | 70.09 | 1022.44 |
| BI689648 | 10 | MAD | 233.64 | 3408.12 |
| BI689648 | 30 | MAD | 700.93 | 10224.35 |
| Dexfadrostat | 0.5 | SAD | 4.23 | 20.20 |
| Dexfadrostat | 1 | SAD | 8.45 | 40.40 |
| Dexfadrostat | 3 | SAD | 25.35 | 121.21 |
| Dexfadrostat | 10 | SAD | 84.52 | 404.04 |
| Dexfadrostat | 30 | SAD | 253.55 | 1212.12 |
| Dexfadrostat | 0.5 | MAD | 4.58 | 25.24 |
| Dexfadrostat | 1 | MAD | 9.15 | 50.49 |
| Dexfadrostat | 3 | MAD | 27.45 | 151.46 |
| Dexfadrostat | 10 | MAD | 91.51 | 504.85 |
| Dexfadrostat | 30 | MAD | 274.53 | 1514.56 |
| LCI699 | 0.5 | SAD | 5.64 | 17.58 |
| LCI699 | 1 | SAD | 11.29 | 35.16 |
| LCI699 | 3 | SAD | 33.87 | 105.49 |
| LCI699 | 10 | SAD | 112.89 | 351.63 |
| LCI699 | 30 | SAD | 338.67 | 1054.90 |
| LCI699 | 0.5 | MAD | 5.79 | 19.44 |
| LCI699 | 1 | MAD | 11.58 | 38.87 |
| LCI699 | 3 | MAD | 34.73 | 116.62 |
| LCI699 | 10 | MAD | 115.76 | 388.75 |
| LCI699 | 30 | MAD | 347.29 | 1166.25 |
| Lorundrostat | 5 | SAD | 31.28 | 271.54 |
| Lorundrostat | 10 | SAD | 62.55 | 543.09 |
| Lorundrostat | 20 | SAD | 125.11 | 1086.17 |
| Lorundrostat | 50 | SAD | 312.76 | 2715.43 |
| Lorundrostat | 100 | SAD | 625.53 | 5430.85 |
| Lorundrostat | 3 | MAD | 23.53 | 241.46 |
| Lorundrostat | 12.5 | MAD | 98.04 | 1006.08 |
| Lorundrostat | 50 | MAD | 392.17 | 4024.33 |
| Lorundrostat | 100 | MAD | 784.34 | 8048.65 |
